# A mixed indium–iron lithium diphosphate, In_0.51_Fe_0.49_LiP_2_O_7_

**DOI:** 10.1107/S1600536811009111

**Published:** 2011-03-15

**Authors:** Hafid Zouihri, Mohamed Saadi, Boujemaa Jaber, Lehcen El Ammari

**Affiliations:** a Laboratoire de Chimie du Solide Appliquée, Faculté des Sciences, Université Mohammed V-Agdal, Avenue Ibn Batouta, BP 1014 Rabat, Morocco; b Centre National pour la Recherche Scientifique et Technique, Division UATRS, Angle Allal AlFassi et Avenue des FAR, Hay Ryad, BP 8027 Rabat, Morocco

## Abstract

The structure of In_0.51_Fe_0.49_LiP_2_O_7_ consists of a three-dimensional network constructed from (In^III^/Fe^III^)O_6_ octa­hedra and P_2_O_7_ groups. Each *M* ^III^O_6_ octa­hedron is linked to six PO_4_ tetra­hedra belonging to five different P_2_O_7_ groups and shares two corners with the same P_2_O_7_ group so as to build infinite chains or rather parallel colums of [*M* ^III^P_2_O_11_] running along the *a* axis. The linkage between these chains or columns defines hepta­gonal tunnels parallel to [100] in which the Li^+^ ions are located in off-centred positions. The In_0.51_Fe_0.49_LiP_2_O_7_ compound can be regarded as one composition of the continuous solid solution between LiFeP_2_O_7_ and LiInP_2_O_7_ whose structure is isotypic with the *A* ^I^FeP_2_O_7_ (*A* ^I^ = Na, K, Rb, Cs and Ag) diphosphate family.

## Related literature

For physical properties and potential applications of *A* ^I^ *M* ^III^P_2_O_7_ (*A* ^I^ = Li, Na, K, Rb, Cs and Ag; *M* ^III^ = Al, Ga, Cr, Fe, In, Y) diphosphates, see: Terebilenko *et al.* (2010 ▶); Hizhnyi *et al.* (2008 ▶); Whangbo *et al.* (2004 ▶); Vitins *et al.* (2000 ▶). For isotypic structures, see: Tran Qui *et al.* (1987 ▶); Rousse *et al.* (2002 ▶). For a closely related structure, see: Zouihri *et al.* (2011 ▶). For background to bond-valence analysis, see: Brown & Altermatt (1985 ▶).

## Experimental

### 

#### Crystal data

In_0.51_Fe_0.49_LiP_2_O_7_
*M* *_r_* = 267.10
Monoclinic, 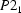

*a* = 4.8698 (2) Å
*b* = 8.2761 (4) Å
*c* = 6.9980 (3) Å
β = 109.650 (2)°
*V* = 265.62 (2) Å^3^
*Z* = 2
Mo *K*α radiation
μ = 4.25 mm^−1^
*T* = 296 K
0.11 × 0.08 × 0.04 mm

#### Data collection

Bruker X8 APEXII CCD area-detector diffractometer
Absorption correction: multi-scan (*SADABS*; Sheldrick, 1999 ▶) *T*_min_ = 0.673, *T*_max_ = 0.845
13153 measured reflections
4262 independent reflections
4200 reflections with *I* > 2σ(*I*)
*R*_int_ = 0.023

#### Refinement

*R*[*F* ^2^ > 2σ(*F* ^2^)] = 0.013
*wR*(*F* ^2^) = 0.032
*S* = 1.04
4262 reflections
103 parameters
2 restraints
Δρ_max_ = 0.74 e Å^−3^
Δρ_min_ = −0.55 e Å^−3^
Absolute structure: Flack (1983 ▶), 1965 Friedel pairs
Flack parameter: 0.021 (6)

### 

Data collection: *APEX2* (Bruker, 2005 ▶); cell refinement: *SAINT* (Bruker, 2005 ▶); data reduction: *SAINT*; program(s) used to solve structure: *SHELXS97* (Sheldrick, 2008 ▶); program(s) used to refine structure: *SHELXL97* (Sheldrick, 2008 ▶); molecular graphics: *ORTEP-3 for Windows* (Farrugia,1997 ▶); software used to prepare material for publication: *WinGX* (Farrugia, 1999 ▶).

## Supplementary Material

Crystal structure: contains datablocks I, global. DOI: 10.1107/S1600536811009111/br2162sup1.cif

Structure factors: contains datablocks I. DOI: 10.1107/S1600536811009111/br2162Isup2.hkl

Additional supplementary materials:  crystallographic information; 3D view; checkCIF report

## Figures and Tables

**Fig. 1.**
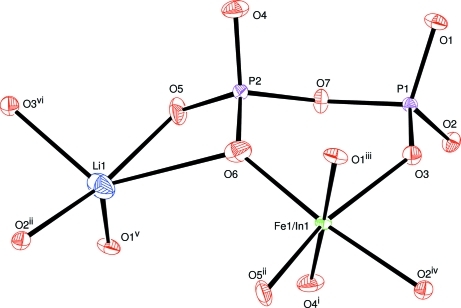
Partial plot of In0.51Fe0.49LiP2O7 crystal structure shawing plyhedra linkage. Displacement ellipsoids are drawn at the 50% probability level. Symmetry codes: (i) x + 1, y, z; (ii) -x, y - 1/2, -z + 1; (iii) -x, y - 1/2, -z + 2; (iv) -x + 1, y - 1/2, -z + 2; (v) -x, y + 1/2, -z + 1; (vi) x, y, z + 1; (vii) -x, y + 1/2, -z + 2; (viii) -x + 1, y + 1/2, -z + 2; (ix) x + 1, y, z + 1; (x) x - 1, y, z; (xi) x, y, z - 1; (xii) x - 1, y, z - 1.

**Fig. 2.**
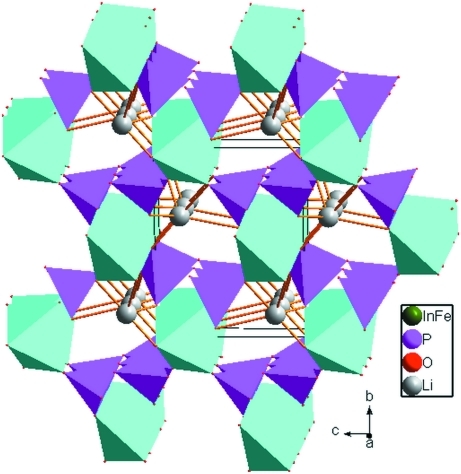
Perspective view along [100] of the In0.51Fe0.49LiP2O7 framework structure showing tunnels where lithium cations are located.

## supplementary crystallographic information

### Comment

As reported in a previous study, physical properties and potential applications of A^I^*M*^III^P_2_O_7_ (A^I^ = Li, Na, K, Rb, Cs and Ag; *M*^III^ = Al, Ga, Cr, Fe, In, Y) diphosphates have attracted the interest of several researchers (Zouihri *et al.* (2011); Terebilenko *et al.* (2010); Hizhnyi *et al.* (2008); Whangbo *et al.* (2004); Vitins *et al.* (2000)). In this context, the present work reports on the determination of In_0.51_Fe_0.49_LiP_2_O_7_ crystal structure from X-ray diffraction single-crystal data.

In an attempt to synthesize an Indium-Iron Lithium Diphosphate, we obtained the following compound of formula: In_0.51_Fe_0.49_LiP_2_O_7_. The calculated valences for the mixed site (In/Fe)^III+^, Li^I+^ and P^V+^ ions are as expected, viz. 3.23, 0.91 and 5.0, respectively. A three-dimensional view of the In_0.51_Fe_0.49_LiP_2_O_7_ crystal structure along the *a* axis, is shown in Fig. 1. The structural network of this phosphate is built up from (In/Fe)O_6_ (to be noted MO_6_) octahedra linked to P_2_O_7_ diphosphate groups by a corner-sharing. The *M*^III^O_6_ octahedra are almost regular with homogeneous *M*^III^—O bond lengths ranging from 2.0225 (9) Å to 2.1230 (6) Å. Each MO_6_ octahedron is surrounded by six PO_4_ tetrahedra belonging to five different P_2_O_7_ groups and shares two corners with the same P_2_O_7_ group as shown in Fig.1 and Fig.2. This induces a 3-D framework in which heptagonal channels parallel to [100] direction are formed. The Li^+^ cations are located in the tunnels but in off-centred positions as shown in Fig.2. Although, the coordination sphere of each Li^+^ cation is composed of four O^2-^ anions located at Li–O distances ranging from 1.956 (3) to 2.107 (3) Å and the fifth at 2.676 (4) Å,in a distorted bi-pyramidal geometry. Furthermore, the diphosphate group exhibits an almost eclipsed conformation with a P–O–P angle of 131.07 (5) °. This value is intermediate between 128.8 (2) ° and 132.7 (4) ° observed for LiFeP_2_0_7_ and LiInP_2_0_7_ respectively) (Rousse *et al.* (2002); Tran Qui *et al.* (1987). This is not surprising because In_0.51_Fe_0.49_LiP_2_O_7_ can be regarded as one composition of the continuous solid solution between LiFeP_2_O_7_ and LiInP_2_O_7_ whose structure is isotypic with the A^I^*M*^III^P_2_O_7_ (A^I^ = Li, Na, K, Rb, Cs and Ag; *M*^III^ = Al, Sc, Cr, Fe, Ga, Y and In) diphosphates family.

### Experimental

Single crystals of the title compound, In_0.51_Fe_0.49_LiP_2_O_7_ phase, were synthesized by flux methods. Indeed, mixture of 0.0004 mole In2O3, 0.0004 mole Fe_2_O_3_, and 0.004 mole (NH_4_)_2_HPO_4_ were addided to 0.0008 mole B(OH)_3_ and 0.0008 mole LiBO_2_ as flux and heated to 1323 K in a platinum crucible. The mixture is lowered to 1223 K with a speed of 0.5°min^-1^ and maintained at this temperature for 20 h and then followed by slow cooling to room temperature at a rate of 0.5°min^-1^ resulted in colourless crystals of the title compound.

### Refinement

The space group is not centro symmetric and the polar axis restraint is generated automatically by Shelxl program. Friedel opposites reflections are not merged. The refinement of the occupancies of the two metal In and Fe and the bond valence sum calculations led to a site occupancy factor of 0.514 (2) for In and 0.486 (2) for Fe. The refinement with a fixed weights (WGHT 0.1) led to a goodness of fit <1 (GooF = S = 0.407). The reflection 002 is omitted because the difference between its calculated and observed intensities is very large. The highest and deepest hole residual peak in the final difference Fourier map are located at 0.60 Å and 0.51 Å, from Fe1.

### Crystal data

**Table:**

In_0.51_Fe_0.49_LiP_2_O_7_	*F*(000) = 254
*M**_r_* = 267.10	*D*_x_ = 3.340 Mg m^−^^3^
Monoclinic, *P*2_1_	Mo *K*α radiation, λ = 0.71073 Å
Hall symbol: P 2yb	Cell parameters from 276 reflections
*a* = 4.8698 (2) Å	θ = 2.4–34.1°
*b* = 8.2761 (4) Å	µ = 4.25 mm^−^^1^
*c* = 6.9980 (3) Å	*T* = 296 K
β = 109.650 (2)°	Prism, colourless
*V* = 265.62 (2) Å^3^	0.11 × 0.08 × 0.04 mm
*Z* = 2	

### Data collection

**Table:**

Bruker X8 APEXII CCD area-detector diffractometer	4262 independent reflections
Radiation source: fine-focus sealed tube	4200 reflections with *I* > 2σ(*I*)
graphite	*R*_int_ = 0.023
ω and φ scans	θ_max_ = 45.0°, θ_min_ = 3.1°
Absorption correction: multi-scan (*SADABS*; Sheldrick, 1999)	*h* = −9→9
*T*_min_ = 0.673, *T*_max_ = 0.845	*k* = −16→16
13153 measured reflections	*l* = −6→13

### Refinement

**Table:**

Refinement on *F*^2^	Secondary atom site location: difference Fourier map
Least-squares matrix: full	*w* = 1/[σ^2^(*F*_o_^2^) + (0.0043*P*)^2^] where *P* = (*F*_o_^2^ + 2*F*_c_^2^)/3
*R*[*F*^2^ > 2σ(*F*^2^)] = 0.013	(Δ/σ)_max_ = 0.003
*wR*(*F*^2^) = 0.032	Δρ_max_ = 0.74 e Å^−^^3^
*S* = 1.04	Δρ_min_ = −0.55 e Å^−^^3^
4262 reflections	Extinction correction: *SHELXL97* (Sheldrick, 2008), Fc^*^=kFc[1+0.001xFc^2^λ^3^/sin(2θ)]^-1/4^
103 parameters	Extinction coefficient: 0.0116 (11)
2 restraints	Absolute structure: Flack (1983), 1965 Friedel pairs
Primary atom site location: structure-invariant direct methods	Flack parameter: 0.021 (6)

### Special details

**Table:**

Geometry. All s.u.'s (except the s.u. in the dihedral angle between two l.s. planes) are estimated using the full covariance matrix. The cell s.u.'s are taken into account individually in the estimation of s.u.'s in distances, angles and torsion angles; correlations between s.u.'s in cell parameters are only used when they are defined by crystal symmetry. An approximate (isotropic) treatment of cell s.u.'s is used for estimating s.u.'s involving l.s. planes.
Refinement. Refinement of *F*^2^ against ALL reflections. The weighted *R*-factor *wR* and goodness of fit *S* are based on *F*^2^, conventional *R*-factors *R* are based on *F*, with *F* set to zero for negative *F*^2^. The threshold expression of *F*^2^ > 2σ(*F*^2^) is used only for calculating *R*-factors(gt) *etc*. and is not relevant to the choice of reflections for refinement. *R*-factors based on *F*^2^ are statistically about twice as large as those based on *F*, and *R*- factors based on ALL data will be even larger.

### Fractional atomic coordinates and isotropic or equivalent isotropic displacement parameters (Å^2^)

**Table:**

	*x*	*y*	*z*	*U*_iso_*/*U*_eq_	Occ. (<1)
In1	0.279204 (12)	0.015329 (8)	0.764879 (9)	0.00719 (2)	0.5138 (15)
Fe1	0.279204 (12)	0.015329 (8)	0.764879 (9)	0.00719 (2)	0.4862 (15)
P1	0.10011 (4)	0.33286 (3)	0.97617 (3)	0.00688 (3)	
P2	−0.29679 (4)	0.23110 (3)	0.58018 (3)	0.00860 (4)	
O1	−0.09909 (15)	0.33270 (9)	1.10229 (11)	0.01212 (10)	
O2	0.30388 (14)	0.47713 (8)	1.01372 (10)	0.01034 (9)	
O3	0.25898 (14)	0.17252 (8)	0.99381 (10)	0.01044 (9)	
O4	−0.56214 (17)	0.19541 (11)	0.63576 (14)	0.01698 (14)	
O5	−0.36828 (19)	0.31921 (11)	0.37968 (11)	0.01694 (14)	
O6	−0.11997 (18)	0.08092 (10)	0.57623 (14)	0.01796 (13)	
O7	−0.09802 (15)	0.35652 (9)	0.74311 (10)	0.01196 (10)	
Li1	−0.3013 (6)	0.1486 (3)	0.1793 (4)	0.0222 (4)	

### Atomic displacement parameters (Å^2^)

**Table:**

	*U*^11^	*U*^22^	*U*^33^	*U*^12^	*U*^13^	*U*^23^
In1	0.00727 (2)	0.00677 (2)	0.00816 (2)	0.00070 (2)	0.00341 (1)	0.00021 (2)
Fe1	0.00727 (2)	0.00677 (2)	0.00816 (2)	0.00070 (2)	0.00341 (1)	0.00021 (2)
P1	0.00698 (6)	0.00674 (7)	0.00732 (7)	−0.00058 (5)	0.00292 (5)	−0.00079 (5)
P2	0.00781 (7)	0.01113 (9)	0.00698 (7)	−0.00074 (6)	0.00265 (5)	−0.00012 (6)
O1	0.0138 (2)	0.0115 (2)	0.0150 (2)	−0.00187 (18)	0.0100 (2)	−0.0021 (2)
O2	0.00973 (19)	0.0089 (2)	0.0116 (2)	−0.00300 (15)	0.00246 (16)	0.00044 (16)
O3	0.0116 (2)	0.0086 (2)	0.0107 (2)	0.00203 (16)	0.00313 (17)	−0.00045 (16)
O4	0.0118 (2)	0.0212 (4)	0.0209 (3)	−0.0004 (2)	0.0093 (2)	0.0037 (3)
O5	0.0205 (3)	0.0217 (4)	0.0069 (2)	−0.0039 (3)	0.0023 (2)	0.0026 (2)
O6	0.0149 (3)	0.0128 (3)	0.0271 (4)	0.0001 (2)	0.0082 (2)	−0.0071 (3)
O7	0.0137 (2)	0.0108 (2)	0.0083 (2)	0.00030 (18)	−0.00045 (17)	−0.00100 (17)
Li1	0.0273 (11)	0.0189 (10)	0.0245 (10)	−0.0051 (8)	0.0140 (8)	−0.0082 (8)

### Geometric parameters (Å, °)

**Table:**

In1—O6	2.0225 (9)	P2—O4	1.4988 (8)
In1—O4^i^	2.0258 (8)	P2—O5	1.5142 (8)
In1—O5^ii^	2.0350 (8)	P2—O6	1.5176 (8)
In1—O3	2.0916 (6)	P2—O7	1.6038 (7)
In1—O1^iii^	2.1133 (7)	Li1—O5	2.092 (2)
In1—O2^iv^	2.1230 (6)	Li1—O6	2.676 (3)
P1—O1	1.5152 (7)	Li1—O2^ii^	1.956 (2)
P1—O2	1.5179 (7)	Li1—O1^v^	1.985 (3)
P1—O3	1.5200 (7)	Li1—O3^vi^	2.107 (3)
P1—O7	1.6034 (7)		
			
O6—In1—O4^i^	86.47 (4)	O2—P1—O7	102.40 (4)
O6—In1—O5^ii^	102.03 (3)	O3—P1—O7	107.75 (4)
O4^i^—In1—O5^ii^	100.84 (3)	O4—P2—O5	112.69 (4)
O6—In1—O3	92.90 (3)	O4—P2—O6	112.79 (5)
O4^i^—In1—O3	90.42 (3)	O5—P2—O6	109.43 (5)
O5^ii^—In1—O3	161.74 (3)	O4—P2—O7	108.06 (5)
O6—In1—O1^iii^	91.67 (3)	O5—P2—O7	104.05 (4)
O4^i^—In1—O1^iii^	177.82 (3)	O6—P2—O7	109.42 (4)
O5^ii^—In1—O1^iii^	80.65 (3)	P1—O7—P2	131.07 (5)
O3—In1—O1^iii^	88.54 (3)	O2^ii^—Li1—O1^v^	104.95 (12)
O6—In1—O2^iv^	171.79 (3)	O2^ii^—Li1—O5	170.56 (15)
O4^i^—In1—O2^iv^	91.17 (3)	O1^v^—Li1—O5	82.36 (10)
O5^ii^—In1—O2^iv^	86.14 (3)	O2^ii^—Li1—O3^vi^	82.74 (10)
O3—In1—O2^iv^	79.25 (2)	O1^v^—Li1—O3^vi^	104.76 (14)
O1^iii^—In1—O2^iv^	90.52 (3)	O5—Li1—O3^vi^	89.67 (10)
O1—P1—O2	114.15 (4)	O2^ii^—Li1—O6	119.23 (13)
O1—P1—O3	111.10 (4)	O1^v^—Li1—O6	114.95 (11)
O2—P1—O3	112.79 (4)	O5—Li1—O6	61.10 (7)
O1—P1—O7	107.97 (4)	O3^vi^—Li1—O6	124.85 (11)

Symmetry codes: (i) *x*+1, *y*, *z*; (ii) −*x*, *y*−1/2, −*z*+1; (iii) −*x*, *y*−1/2, −*z*+2; (iv) −*x*+1, *y*−1/2, −*z*+2; (v) *x*, *y*, *z*−1; (vi) *x*−1, *y*, *z*−1.

## References

[bb1] Brown, I. D. & Altermatt, D. (1985). *Acta Cryst.* B**41**, 244–247.

[bb2] Bruker (2005). *APEX2* and *SAINT* Bruker AXS Inc., Madison, Wisconsin, USA.

[bb3] Farrugia, L. J. (1997). *J. Appl. Cryst.* **30**, 565.

[bb4] Farrugia, L. J. (1999). *J. Appl. Cryst.* **32**, 837–838.

[bb5] Flack, H. D. (1983). *Acta Cryst.* A**39**, 876–881.

[bb6] Hizhnyi, Y. A., Oliynyk, A., Gomenyuk, O., Nedilko, S. G., Nagornyi, P., Bojko, R. & Bojko, V. (2008). *Opt. Mater.* **30**, 687–689.

[bb7] Rousse, G., Rodriguez-Carvajal, J., Wurm, C. & Masquelier, C. (2002). *Solid State Sci.* **4**, 973–978.

[bb8] Sheldrick, G. M. (1999). *SADABS* University of Gottingen, Germany.

[bb9] Sheldrick, G. M. (2008). *Acta Cryst.* A**64**, 112–122. 10.1107/S0108767307043930 18156677

[bb10] Terebilenko, K. V., Kirichok, A. A., Baumer, V. N., Sereduk, M., Slobodyanik, N. S. & Gütlich, P. (2010). *J. Solid State Chem.* **183**, 1473-1476.

[bb11] Tran Qui, D., Hamdoune, S. & Le Page, Y. (1987). *Acta Cryst.* C**43**, 201–202.

[bb12] Vitins, G., Kanepe, Z., Vitins, A., Ronis, J., Dindune, A. & Lusis, A. (2000). *J. Solid State Electochem.* **4**, 146–152.

[bb13] Whangbo, M.-H., Dai, D. & Koo, H.-J. (2004). *Dalton Trans.* pp. 3019–3025. 10.1039/b401312n 15452625

[bb14] Zouihri, H., Saadi, M., Jaber, B. & El Ammari, L. (2011). *Acta Cryst.* E**67**, i2. 10.1107/S1600536810052451 PMC3050398 21522510

